# My Data, My Choice? – German Patient Organizations’ Attitudes towards Big Data-Driven Approaches in Personalized Medicine. An Empirical-Ethical Study

**DOI:** 10.1007/s10916-020-01702-7

**Published:** 2021-02-22

**Authors:** Carolin Martina Rauter, Sabine Wöhlke, Silke Schicktanz

**Affiliations:** 1 Department of Medical Ethics and History of Medicine, University Medical Center Göttingen, Göttingen, Germany; 2 Hamburg University of Applied Science, HAW Hamburg, Germany

**Keywords:** Big data, Personalized medicine, Patient organizations, Stakeholder, Interviews, Empirical ethics

## Abstract

Personalized medicine (PM) operates with biological data to optimize therapy or prevention and to achieve cost reduction. Associated data may consist of large variations of informational subtypes e.g. genetic characteristics and their epigenetic modifications, biomarkers or even individual lifestyle factors. Present innovations in the field of information technology have already enabled the procession of increasingly large amounts of such data (‘volume’) from various sources (‘variety’) and varying quality in terms of data accuracy (‘veracity’) to facilitate the generation and analyzation of messy data sets within a short and highly efficient time period (‘velocity’) to provide insights into previously unknown connections and correlations between different items (‘value’). As such developments are characteristics of Big Data approaches, Big Data itself has become an important catchphrase that is closely linked to the emerging foundations and approaches of PM. However, as ethical concerns have been pointed out by experts in the debate already, moral concerns by stakeholders such as patient organizations (POs) need to be reflected in this context as well. We used an empirical-ethical approach including a website-analysis and 27 telephone-interviews for gaining in-depth insight into German POs’ perspectives on PM and Big Data. Our results show that not all POs are stakeholders in the same way. Comparing the perspectives and political engagement of the minority of POs that is currently actively involved in research around PM and Big Data-driven research led to four stakeholder sub-classifications: ‘mediators’ support research projects through facilitating researcher’s access to the patient community while simultaneously selecting projects they preferably support while ‘*cooperators*’ tend to contribute more directly to research projects by providing and implemeting patient perspectives. ‘*Financers*’ provide financial resources. ‘*Independents*’ keep control over their collected samples and associated patient-related information with a strong interest in making autonomous decisions about its scientific use. A more detailed terminology for the involvement of POs as stakeholders facilitates the adressing of their aims and goals. Based on our results, the ‘*independents*’ subgroup is a promising candidate for future collaborations in scientific research. Additionally, we identified gaps in PO’s knowledge about PM and Big Data. Based on these findings, approaches can be developed to increase data and statistical literacy. This way, the full potential of stakeholder involvement of POs can be made accessible in discourses around PM and Big Data.

**Supplementary Information:**

The online version contains supplementary material available at 10.1007/s10916-020-01702-7.

## Introduction

Patient-centeredness in research and medical care has become an important goal in both international and German health policy dealing with the future-shaping of medical care [1–3]. It is driven by current and future developments which aim to optimize prevention and therapy (‘personalized medicine’^1^) by facilitating an ever more precise stratification of patients into subgroups based on bio-physiological characteristics and associated lifestyle information. This approach depends on the generation, usage and linking of large amounts of data, ranging from genetic information and tissue biomarkers up to structured information such as (electronic) patient records or patient registers. This phenomenon is commonly referred to as biomedical ‘Big Data’^2^ [4]. In this context, patients and patient organizations (POs) form a crucially important stakeholder group^3^Their data, along with the associated ethical concerns regarding informed consent, privacy, data ownership, as well as epistemological aspects and issues around the ‘Big Data Divide’^4^ [11] are the drivers of future progress in this field. POs may vary strongly regarding their characteristics such as member structure, size or interaction with other stakeholders from health policy, science or industry. On a very general level we define POs as collective actors who aim to collectively advocate for patient interests. Many of them are led by patients themselves or by caregivers [13].

When it comes to assessing the opportunities and risks of PM and biomedical Big Data-driven research, POs have become increasingly active stakeholders [9, 14]. While traditionally POs in Germany were seen merely as self-help groups [15], international POs now collect research data and even seek to shape research projects [16–18] in a similar way like other stakeholders such as researchers and their associated institutions or companies pursuing commercial interests. Most remarkably, patients have sought to shift paradigms about expertise in research by designing and implementing research projects themselves [19]. Especially patients with rare diseases can benefit from networking and data sharing because they face smaller numbers of experts and long journeys for treatment. An increase in data-sharing can help overcome these problems by facilitating recruitment of participants worldwide and bundling international expert knowledge [20]. Some international POs for rare diseases are already remarkably active and innovative contributors to research projects focusing on their diseases [21].

The debate about patient involvement in personalized medicine and biomedical Big Data is still predominantly shaped by experts [22]. Currently, there is a lack of more precise knowledge regarding the involvement and attitudes of POs in Germany in this context. Therefore, our study aims to answer the following questions: Which opportunities and risks do POs in Germany see in PM approaches such as genetic and non-genetic tests and associated biomedical Big Data-based research projects and how do they make use of their stakeholder positions as representatives of patient interests in this context?

## Methods

**Fig. 1:**
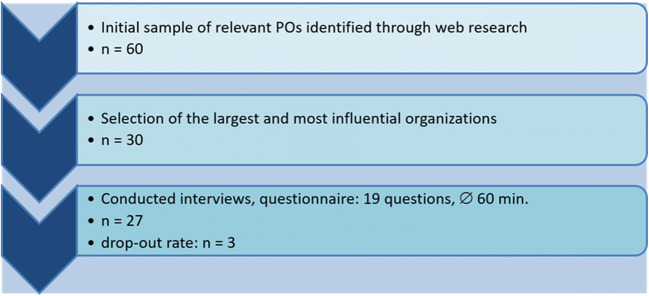
Simplified description of sample creation

For our research, we used an empirical-ethical approach^5^ [23] based on a qualitative mixed-methods framework. Our empirical methods consisted of first, a website analysis [25] of POs websites (see ESM 1). Second, we conducted and analyzed 27 semi-structured expert interviews on the telephone [26] with representatives from German POs (see Fig. 1 for qualitative research workflow and ESM 2 for interview guidelines).^6^ We applied a qualitative content analysis [28] of the interview transcripts using the software ATLAS.ti©.

Based on this definition, we used the public databases of the German Contact and Information Point for the Initiation and Support of Self-Help Groups (NAKOS)^7^ to identify relevant POs. The initial sample of 60 POs was used for a website analysis from which we selected 30 organizations as potential interview partners. We chose organizations based on size (those with the largest number of members) and political influence (e. g. those that claimed involvement in health policy) (for more detailed results, see ESM 1). Twenty-seven organizations and their associated representatives agreed to participate in our study.

A semi-structured interview guideline was developed using ethical themes and statements from leading position papers [29]. Interviews were conducted by three researchers and ranged between 26 to 86 min in length (60 min average). When new arguments no longer emerged, we judged that saturation had been reached [30] and we ended participant recruitment after interview no. 27. All interviews were transcribed and anonymized. During coding, inductive codes were added and re-applied on the already coded material. After coding, ideal types [31] of POs were developed from both the website analysis results and the coded interview quotes to characterize the approaches to these topics of different POs [32]. When quantifying results, we used the same proportions as Schaper et al. (2019)^8^ [33].

### Limitations

Our research also had some limitations. First, when selecting POs for our interviews we relied on information from their websites which might change over time due to updates etc. and which might not be fully available to future readers. To make this information level traceable, we documented our findings in the electronic supplementary material attached (see ESM 1, ESM 3). Second, some characteristics of POs may be specific to Germany, where self-help oriented associations with a local scope dominate [34]. However, our sample predominantly covers national associations and can therefore not be considered representative of the German PO landscape as such. Our typology may serve as a framework for further investigation in this field.

Third, because there have been so few studies on the views of POs regarding PM and biomedical Big Data, our focus in the interviews was very general and allowed interviewees to take very subjective positions. Therefore, the typology is based on their most outstanding features and might need to be further specified for POs in countries where the debate is more developed.

## Results

**Fig. 2:**
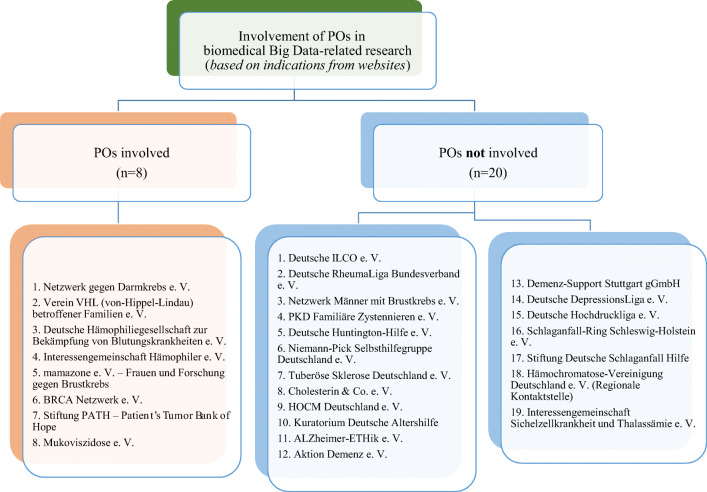
Involvement of POs in biomedical Big Data-related research (*based on indications from websites*)

**Fig. 3:**
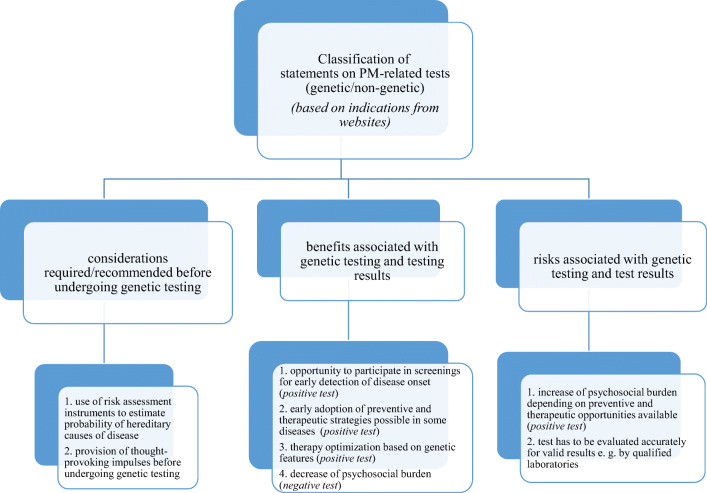
Classification of statements on PM-related tests (genetic/non-genetic) *(based on indications from websites)*

Our results show that roughly one third (*n* = 11) of POs in our sample indicated some involvement in PM-related biomedical Big Data projects on their websites (see Fig. 2), usually forms of health-political involvement (ESM 1). When POs addressed opportunities and risks of genetic testing online, weighed arguments were given that highlight the importance of careful individual decision-making around getting tested (ESM 3, Fig. 3 and Table 3).

### POs perspectives on opportunities and risks associated with PM and biomedical big data-driven research

Perspectives concerning genetic testing provided on PO websites consisted of statements about opportunities and risks as well as recommendations e.g. under-going genetic counselling (see Fig. 3). Some organizations recommended prior risk assessment before having a test to estimate the risk of having a hereditary predisposition (e. g. questionnaire for risk assessment provided by ILCO,^9^ drawing of a family tree if a hereditary component in disease is suspected, as recommended by Cholesterin & Co.^10^). Another PO provides users contemplating genetic testing with a unique online catalogue of thought-provoking statements^11^ covering a broad range of psychosocial issues, such as motivation for testing, impact on partnership, family planning, education and profession, as well as the impact of testing on partners and family members, friends and employer. During the interviews, interviewees also referred to biomarkers, gene therapy and pre-implantation genetic diagnosis. Interestingly, while references to genetic tests and their implications were addressed on PO’s websites (ESM 1) some representatives of POs reported that this issue was not frequently discussed within their organization (quot. 1–3, ESM 4). Uncertainty regarding the meaning of PM was also expressed when the implementation of PM-approaches was perceived to be far in the future (quot. 4–6, ESM 4). Positive attitudes towards PM-related approaches included opportunities for early detection and prevention as well as finding the right treatment (quot. 7–8, ESM 4). Skeptical attitudes were expressed when results of genetic testing did not lead to therapeutic consequences (quot. 9, ESM 4). Additionally, the risk of psychosocial burden when learning about test results and possible violations of the right not to know were addressed as critical consequences (quot. 10–11, ESM 4). Furthermore, the validity of tests was considered to be crucial (quot. 12, ESM 4). When taking other stakeholders into consideration, interviewees raised the fear of being disadvantaged, e.g. by insurance companies and employers due to test results (quot. 13, 14, ESM 4). When referring to biomedical Big Data, some interviewees struggled with the terminology and asked for a definition. However, most interviewees were aware of the linkages between biomedical Big Data and the implementation of PM-approaches (quot. 15–16, ESM 4). Roughly a quarter of the POs saw the ability to tailor diagnostic procedures and therapies more precisely to patient needs as one big promise and advantage of Big Data (quot. 17–18, ESM 4). The second-most cited advantage was the opportunity to monitor risk factors, leading to more detailed insights on how to refine preventative approaches and to improve medical care (quot. 19–20, ESM 4). Additionally, Big Data was associated with improvements in documentary processes for individual disease management and facilitating the work of doctors (quot. 21–22, ESM 4). A less common perspective referred to data serving as evidence in case of patient claims for compensation (quot. 23, ESM 4). Furthermore, we noticed with interest that a minority of interviewees (*n* = 4) raised the issue of self-tracking during in the interview (quot. 24–28, ESM 4), which they stated was based on either on their experiences as individuals or their knowledge about ongoing projects. Therefore, we can assume that at least some interviewees are aware of fitness tracking becoming a more and more commonly used Big Data-associated technology. One interviewee raised the idea of data donation (quot. 29, ESM 4). Regarding potential harms of biomedical Big Data and associated research, interviewees raised great concerns, such as fear of discrimination and a general skeptical attitude towards other stakeholders involved in Big Data projects. Particular criticism was directed at companies and organizations with commercial interests, such as Facebook, Google and others (quot. 30, ESM 4). In this context, a feeling of losing control over data was widely expressed, and it was emphasized that a lack of transparency can lead to the misuse of data (quot. 31, ESM 4). Interestingly, some interviewees also referred to the danger of instrumentalization of data in different political contexts (quot. 32, ESM 4). In general, although most interviewees provided well-weighed opinions about both PM and biomedical Big Data and referred to opportunities as well as risks when talking about them (quot. 33, ESM 4), we noticed that overall attitudes can be described as rather critical and observant. Furthermore, various conditions and claims regarding PM, biomedical Big Data and associated research coincided with attitudes which we will be elaborating in the next section.

### POs perspectives on requirements for the implementation of PM and biomedical big data-driven research

Most interviewees claimed that patients should be regarded as keepers of their medical data (quot. 34, ESM 4). Even when sharing data in selected contexts was considered to be beneficial (e. g. data sharing with doctors or researchers), some interviewees were in favor of authorization concepts for patients that would allow them to specify which kinds of data they wanted to share or hold back (quot. 34, ESM 4). Additionally, it was emphasized that certain situations were inappropriate for asking patients to participate in research, e. g. after receiving a diagnosis causing psychological and emotional stress (quot. 35, ESM 4). When using medical data for research, interviewees desired sufficient, concrete information about the sort of data being used and the aims of the research project for which it is used (quot. 31, ESM 4). Furthermore, they stressed the importance of properly anonymizing data (quot. 36, ESM 4), which they saw as a crucial precondition for consent to participate in data-intense research (quot. 37, ESM 4). However, regarding rare diseases, some interviewees noted the limitations to anonymizing data because tracing back even anonymized data to individuals was considered to be technically feasible in these cases (quot. 38, ESM 4). In general, ensuring data protection was seen to be of great importance (quot. 39, ESM 4). However, when addressing data security on a more general level, one interviewee points out to risks that will always remain when working with data as there is no such thing as being 100% safety against to hacking attacks (quot. 40, ESM 4). Aside from requirements around privacy data security, interviewees also addressed the need to improve data infrastructure. Furthermore, improvements regarding interface management were discussed (quot. 41, ESM 4).

### Differences in approaches of POs regarding contribution to and involvement in research around PM and biomedical big data

Based on our interview material, we were able to construct a typology of four approaches of POs different contributions to and involvement in research on PM and biomedical Big Data. We refer to involved POs as *mediators, cooperators, financers and independents.*^12^ POs that did not fit into these four categories, were assigned to a fifth group. It characterizes those POs that did not describe themselves as particulary interested or involved in promoting research in the fields of PM and Big Data so we introduced a fifth label called ‘*alternatives*’. These five categories serve as sociological ideal types [31] to reduce the complexity we found in our empirical findings for the purposes of comparison, meaning that most POs tend to meet the characteristics of more than one proto-typological approach.

#### Mediators

*Mediators* support research on PM and biomedical Big Data by cooperating with researchers on two levels. They circulate information on ongoing studies within their patient community (e.g. by publishing advertisements for studies on their websites,^13^ see also quot. 42, ESM 4) and thus facilitate participant recruitment for research. Mobilized individuals can contribute to research by providing either requested biomaterial (e. g. tumor tissue, blood etc.) and/or other information considered to be relevant for research purposes (e.g. sociodemographic data, indications around patient related outcomes etc.). This approach creates a mutually beneficial situation: researchers receive facilitated access to their target group and to useful biomaterial and/or data, while POs can select projects they want to advertise based on their preferences and interests. Furthermore, patients can individually decide to which studies and projects they want to contribute, or if they even want to contribute at all. However, we also identified a case in which doctors were legally obligated to forward health-related information of their patients to a patient register.^14^ By promoting projects and studies they are particularly interested in, *mediators* may have the capacities to indirectly influence research agendas by facilitating or complicating researcher’s access to patients as their ‘target group’.

#### Cooperators

Compared to the group of *mediators*, *cooperators* are more actively engaged in relationships with other stakeholders working in the field of PM and Big Data. These organizations are on variable levels involved in setting agendas or providing perspectives and advice for research and clinical practice. Their role is often associated with representing ‘the patient view’ on certain aspects in the research process or the process of clinical implementation. However, ‘involvement’ may cover a broad spectrum of interactions in need for a more precise critical ethical assessment of e.g. hierarchies or power asymmetries that might be a limiting factor to patient’s standing and influence.

#### Financers

*Financers* support research through fundraising (quot. 43, ESM 4). Aside from financial support for research projects e.g. research grants tendered by foundations, we also identified offers such as research prizes or even scholarships for researchers.^15^ However, this approach was comparatively rare in our sample, most likely because the financial resources of patient-led POs are limited (quot. 44, 45, ESM 4).

#### Independent

The *independent* are characterized by their aim to generate and administer data for scientific purposes themselves. In their most sophisticated form, they have established PM- and biomedical Big Data-related projects such as patient registries and biobanks (quot. 46, 47, ESM 4). These organizations are of tremendous interest to researchers, who often request their cooperation (quot. 48, ESM 4). Others have described the execution of smaller projects, such as surveys, that are distributed among their members to gain insight into topics of interest for the organization (quot. 49, ESM 4). Interestingly, an associated interviewee referred to the term ‘power’ in this context by explaining that insights from data can lead to valuable arguments in discourses that might strengthen the position of patients (quot. 50, ESM 4).

#### Alternatives

Aside from these approaches, some POs also explained that involvement in discussions and/or research concerning PM and biomedical Big Data is not an essential part of their work. These organizations often refer to their work as ‘self-help’ (quot. 51, ESM 4), explaining that for them, raising awareness about the disease as well as supporting affected people, their family members and caregivers is very important. Particularly POs advocating for diseases such as dementia, tended to focus on a more self-help and community-oriented approach. We summarize these POs by referring to them as ‘*alternatives*’ as from their point of view ‘alternative’ approaches of biomedical perspectives tend to be neglected by politics or sciences regardint the improvement of the life of certain patient groups, their relatives and caregivers.

Within our typology, we identified one PO to be most active in public health-related issues. Varying levels of satisfaction were expressed regarding current research-related cooperations. POs were more likely to be satisfied if they felt that cooperation between researchers and patients was conducted on an equal eye-level basis (quot. 52, ESM 4). However, individual POs also expressed dissatisfaction with cooperations, particularly when they felt that their arguments had been neglected or if they considered the implementation of their perspectives to be not sustainable (quot. 53, ESM 4) (see Table 1).

**Table 1:** Assignment of interviewed POs to the typology. As the majority of the interviewed POs usually fulfilled more than only one typological characteristic, we highlighted the most outstanding position

	Mediators	Cooperators	Financers	Indepedent	Alternatives
Deutsche ILCO e. V.		X			**X**
Netzwerk gegen Darmkrebs e. V.		**X**	X		
Verein VHL (von-Hippel-Lindau) betroffener Familien e. V.	X			**X**	
Deutsche Hämophiliegesellschaft zur Bekämpfung von Blutungskrankheiten e. V.	X	**X**			
Interessengemeinschaft Hämophiler e. V.	X	**X**			
Deutsche RheumaLiga Bundesverband e. V.	X	**X**	X		
mamazone e. V. – Frauen und Forschung gegen Brustkrebs	X	X		**X**	
BRCA Netzwerk e. V.	**X**				
Stiftung PATH – Patient’s Tumor Bank of Hope		X		**X**	
Netzwerk Männer mit Brustkrebs e. V.	X	**X**			
PKD Familiäre Zystennieren e. V.	X	**X**			
Deutsche Huntington-Hilfe e. V.	X				
Niemann-Pick Selbsthilfegruppe Deutschland e. V.	X				
Tuberöse Sklerose Deutschland e. V.		**X**	X		
Cholesterin & Co. e. V.		X			X
HOCM Deutschland e. V.		X			
Mukoviszidose e. V.	X	X	X	**X**	
Kuratorium Deutsche Altershilfe		X			
ALZheimer-ETHik e. V.					X
Aktion Demenz e. V.					X
Demenz-Support Stuttgart gGmbH		X			
Deutsche DepressionsLiga e. V.	**X**	X			
Deutsche Hochdruckliga e. V.		X			
Schlaganfall-Ring Schleswig-Holstein e. V.		X			
Stiftung Deutsche Schlaganfall Hilfe		X			
Hämochomatose-Vereinigung Deutschland e. V. (Regionale Kontaktstelle)		X			**X**
Interessengemeinschaft Sichelzellkrankheit und Thalassämie e. V.		X		X	**X**

## Discussion

In our introduction, we stressed the ethical relevance of patients and POs as stakeholders in shaping the approaches of PM and biomedical Big Data. Based on our analysis of PO websites and interview data, we were able to develop a typology of four PO strategies for shaping PM- and biomedical Big Data-related approaches. While the level of engagement of POs varied significantly, we can draw some general and also nuanced patterns regarding the perception and assessment of opportunities and risks.

Overall, the general attitude of the interviewed POs remained rather skeptical. Context relatedness of PM and biomedical Big Data was stressed to be very important when it came to evaluating particular applications in both fields. Because contexts are not exclusively shaped by patients’ interests but also by those of other stakeholders involved in discussions about PM and biomedical Big Data, the relationship between POs and other third parties was identified to be a central issue. We were able to identify ethical challenges on two levels: *barriers to the recognition of patients as partners* and *structural inequalities between patients and other stakeholders*.

### Barriers to the recognition of patients as partners

According to Beier et al. (2019) [35], several ethical preconditions must be fulfilled for research involving patients to be considered truely participatory. First, it should be critically reflected whether involving patients mainly serves as an instrument, e.g. to facilitate data collection, or whether it will improve research by taking patient perspectives into consideration. When taking into consideration the approaches of '*mediators*' and '*financers*', patients provide useful resources to researchers. However, these approaches alone are unlikely to establish a truly equitable relationship between researchers and patients. If co-shaping research projects is neither encouraged nor desired, patients remain passive and limited in their opportunities to contribute. In terms of ethical concepts and values such as autonomy and trust, patient’s capabilities to act autonomously are not only limited but explicitly violated when taking a Kantian perspective on asymmetrical relationships between patients and other experts [36]. Because representatives of POs in our sample have addressed concerns and distrust about data being used by commercially oriented companies and other third-party stakeholders, we perceive this as an indication that POs may not yet have been willing and/or able to successfully represent patients’ arguments and concerns. This may also be due to a lack of resources that patients need to become involved, e.g. access to funding and sources of knowledge [35], or competencies such as digital literacy [37]. Because most German POs have their roots in the environment of self-help, pre-existing stereotypes of patients being passive and in need of expert advice might lead to an ethically unjustified pre-exclusion of their arguments [22].

### Structural inequalities between patients and other stakeholders

Based on our findings, POs participating in our study do not generally have at their disposal the same level of knowledge and skills concerning the generation, processing and interpretation of data, as well as the technology required, compared to other stakeholders from the fields of research or biotechnological industry. Generally speaking, how they treat other stakeholders is based on a general distinction between those they do or do not trust (e.g. making health-related data accessible for stakeholder x but not for stakeholder y). On the one hand, this may be a result of a PO’s individual priorities (e.g. prioritizing self-help oriented approaches to support patients and their relatives/caregivers over research and health care involvement). On the other hand, it could also indicate a lack of capacity and competence to autonomously work with data. Based on our results, not all POs had heard of the term Big Data before, with some interviewees asking for a definition during the interview. Therefore, due to a lack of precise knowledge as well as access to associated technology, patients and POs are likely to be at risk of being affected by the abovementioned ‘Big Data Divide’ [11]. In most unfavorable cases, this may lead not only to fundamental inequalities in the capacity of POs to contribute to and be involved in research concerning PM and biomedical Big Data compared to other stakeholders, but also their ability to defend their claims to privacy and ensure their informational self-determination.^16^

## Conclusion and outlook

Patient-centeredness as well as patient involvement in research and care are internationally promoted in many policy papers. Our results indicate that only a small number of POs can be considered to be involved at eye level with other stakeholders regarding their contribution to related PM- and biomedical Big Data approaches as well as to the policy discourse. This points to the need to find solutions to sensitize or to empower rather ‘passive’ POs to be engaged with this important change of medical research and health care systems. As our results also indicate, digital literacy might be key issue for POs and general public to be involved [37]. Therefore, information about PM and Big Data-driven research should be explained and communicated in everyday language by experts from the fields of biomedical sciences and by public administration that promotes such developments. This is yet often not sufficiently happening. A better understanding of its general implications for society, health care systems and future allocation decisions seems to be necessary to generate an informed public debate that involves all types of stakeholders. Our typology (*‘mediators’, ‘cooperators’, ‘financers’, ‘independents’* and 'alternatives') can help to reflect on the diverse needs POs have and roles POs can play according to their specific expertise and interests.

Additionally, terms such as ‘involvement’ or ‘participation’ consist of various normative dimensions, ranging from simple opt-in consent in research participation to ambitious forms of political, co-decision making [35] - a diverse spectrum we also identified in our typology of '*cooperators*'. Ideally, patients should be encouraged to discuss with researchers at eye level and to participate in the execution of PM- and biomedical Big Data-related projects at all steps of the process. Furthermore, we believe that intercultural research between POs in different countries might provide more detailed insights into cultural and historical aspects shaping the involvement of patients in research projects and shaping research themselves by conducting their own projects.

Subsequently, we like to point out, that more PO involvement does not make all PM- and Big Data approaches ‘ethically acceptable’. Ethical acceptance would also need to include considerations of protecting human rights, of justice and fairness, and of fair benefit for various social groups, beyond those of POs, e. g. of the public, the next generations, and implications for research and health care.

## Supplementary Information

ESM 1 (DOCX 38.5 kb)

ESM 2 (DOCX 491 kb)

ESM 3 (DOCX 56.8 kb)

ESM 4 (DOCX 57.6 kb)

## Footnotes

1 The term personalized medicine can mean different things [5, 6]. We refer here to the following definition: ‘PM seeks to improve tailoring and timing of preventive and therapeutic measures by utilizing biological information and biomarkers on the level of molecular disease pathways, genetics, proteomics as well as metabolomics.’ [5].

2 Big Data also varies in its definition but is generally characterized by volume (data of intense breadth and/or depth), velocity (frequency of data generation, delivery and procession), variety (integration of data from various sources) and veracity (validity and accuracy of data) in the context of health care [7].

3 Here, the term ‘stakeholder’ refers to individuals and groups who have an interest (‘a stake’) in a certain bio- or health-political issue due to their contextual affectedness [8]. Patients aim to become more involved in discussions on personalized medicine and the associated use of data [9]. Consequently, it can be argued that the active involvement of patients’ voices in health-political discussions is crucial to ensure a fair, balanced discourse. Their input and deliberation between stakeholders are essential for the development of socially robust solutions, e. g. by an intensified discourse between scientists and ‘expert patients’ [10].

4 The term ‘Big Data Divide’ refers to inequalities between providers of data and those who are capable of analyzing it due to their access to required technology and/or knowledge [11]. This asymmetry is ethically problematic because it risks social sorting and violates individual autonomy [12].

5 Empirical ethics allows us to identify relevant moral issues and assumptions stated by POs in a context-maintaining way by using methods from social sciences [23, 24]. This approach could be used to make descriptive statements about patients’ perspectives towards personalized medicine, biomedical Big Data and associated research projects, but it simultaneously provides us with insights into the factors shaping these perspectives.

6 We refer to representatives of POs as experts due to their access to inside knowledge around patient-related issues and perspectives when discussing personalized medicine and Big Data as the analysis of expert knowledge does not only entail only professional knowledge but also includes active participants in community affairs, self-help groups, as well as volunteers in welfare [27].

7 NAKOS – Nationale Kontakt- und Informationsstelle zur Anregung und Unterstützung von Selbsthilfegruppen (own translation), https://www.nakos.de/adressen/gruen/. Accessed 29 Nov 2020.

8 “(…) The reporting of participants’ positions follows the scheme: many/most = > 50%, some = 10–50%, few = 0–10% of participants. “Majority” refers to absolute majority unless otherwise specified while “minority” means a very small number of participants. (…)” [33].

9 https://www.ilco.de/fileadmin/user_upload/Erblicher_DK_Frageboegen/fragebogen-erblicher-darmkrebs-englisch.pdf. Accessed 29 Nov 2020.

10 https://cholco.org/wp-content/downloads/20120229-Stammbaum.pdf. Accessed 29 Nov 2020. Available in German only.

11 https://dhh-ev.de/sites/default/files/Literatur/Denkanstoesse.pdf . Accessed 29 Nov 2020. Available in German only. Own translation of topics raised.

12 Michaela Mayrhofer’s work on European POs‘invovlement in biobanking activities resulted in a similar typology. She distinguishes POs activities based on the following typology: *interface types* („Schnittstellentyp“, own translation), *initiazing types* („Initiatortyp“, own translation) and *do-it-yourself type*. https://www.gen-ethisches-netzwerk.de/patientenorganisationen-und-biobanken Accessed 04 Oct 2020

13 The following POs provide a section for calls for participation in ongoing research projects on their websites: https://www.brca-netzwerk.de/forschungsprojekte/;https://www.brustkrebs-beim-mann.de/studien/;http://www.pkdcure.de/index.php?page=aktuelle-studien;https://www.depressionsliga.de/studien_umfragen.html. Accessed 29 Nov 2020. Available in German only.

14 Based on legal regulations in Germany (German Transfusion Act), the German Hemophilia Registry was established. For further information see also https://www.pei.de/EN/information/pharmacists-physicians/dhr-german-haemophilia-registry/objectives/dhr-objectives-node.html. Accessed 20 Oct 2019.

15 See for example Tuberöse Sklerose Deutschland e. V. and its offers to researchers and doctors: https://www.tsdev.org/leben-mit-tsc/wissenschaft-forschung/. Accessed 29 Nov 2020. Available in German only.

16 “What the expression ‘informational self-determination’ means is rather that an individual’s control over the data and information produced about him is a (necessary but insufficient) precondition for him to live an existence that may be said ‘self-determined’. This is an important thing to recall today, as personal data (genetic and/or digital) have become proxies for persons with the intensification of governmental ‘identity projects’.” [38].
