# Supplementary material for: My Data, My Choice? – German Patient Organizations’ Attitudes towards Big Data-Driven Approaches in Personalized Medicine. An Empirical-Ethical Study
Source: J Med Syst. 2021 Feb 22;45(4):43. doi: 10.1007/s10916-020-01702-7 (PMC7900081; doi:10.1007/s10916-020-01702-7)
Supplement: Supplementary file 1 — (DOCX 38.5 kb) [file 10916_2020_1702_MOESM1_ESM.docx]

ESM 1: Table 2: Website analysis on patient organization’s (POs) positions and perspectives towards personalized medicine and Big Data (carried out between 08/2018 and 01/2019)

Title: My data, my choice? – German patient organizations’ attitudes towards Big Data-driven approaches in personalized medicine. An empirical-ethical study.

Journal: Journal of Medical Systems

Authors: Carolin Martina Rauter^[[1]](#footnote-1)^, Sabine Wöhlke, Silke Schicktanz

Affilation: Institute for Medical Ethics and History of Medicine, University Medical Center Göttingen, Humboldtallee 36, 37073 Göttingen

|  | Patient Organization´ | Focus | Member-ship size^[[2]](#footnote-2)^* | Main goals of the organization with relevance for our research purposes | Activities in health policy | Information on genetic testing or biomarker diagnostics | Indications of Big Data-related activities |
| --- | --- | --- | --- | --- | --- | --- | --- |
| 1 | Deutsche ILCO e. V.  https://www.ilco.de/ | Ostomy and Bowel Cancer Patients | 7.100 | Patient advocacy, supporting research and prevention | Yes | Indication of benefits of genetic test results, questionnaire for risk assessment of hereditary bowel cancer available on website | - |
| 2 | Netzwerk gegen Darmkrebs e. V.  https://www.netzwerk-gegen-darmkrebs.de/ | Bowel Cancer | 222 | Support of research on prevention, identification and therapy of bowel cancer | Yes | Possible hereditary components of disease are indicated.  References to stool biomarkers | Participation in a German-Czech research cooperation on a notably increase of bowel cancer in Upper Franconia and the Czech Republic |
| 3 | Verein VHL (von-Hippel-Lindau) betroffener Familien e. V.  http://www.hippel-lindau.de/ | Von-Hippel-Lindau-Syndrome | 260 | Support of research in diagnostic and therapy | Yes | Indication of benefits of genetic test results, elaborations on inheritance of the disease and availability of genetic testing opportunities, references to accredited laboratories offering tests and participating in quality controls on a regular basis | Establishment of a biobank at the Dr. Margarete Fischer-Bosch-Institute of Clinical Pharmacology (IKP) in Stuttgart  Establishment of a patient register |
| 4 | Deutsche Hämophiliegesellschaft zur Bekämpfung von Blutungskrankheiten e. V.  https://www.dhg.de/ | Bleeding Diseases | 2.300 | Support of research and medical care | Yes | Information on hereditary aspects of bleeding diseases available when scrolling through subsections of specific diseases | Participation in the establishment of the German Hemophilia Registry located at the Paul-Ehrlich-Institut |
| 5 | Interessengemeinschaft Hämophiler e. V.  https://www.igh.info/ | Hemophilia | 950 | Support of research projects, assessing future perspectives for therapy | Yes | Information on hereditary aspects of hemophilia available on website, references to the opportunity of genetic testing for the identification of female carriers of the disease-associated gene, references to the opportunity of prenatal testing | Participation in the establishment of the German Hemophilia Registry located at the Paul-Ehrlich-Institut |
| 6 | Deutsche RheumaLiga Bundesverband e. V.  https://www.rheuma-liga.de/startseite/ | Rheumatism | 300.000 | Representation of patient’s interests, research support | Yes | References to the role of biomarkers in rheumatic diseases | - |
| 7 | mamazone e. V. – Frauen und Forschung gegen Brustkrebs  https://www.mamazone.de/ | Breast Cancer | 1800 | Supporting informed and self-determined patients, supporting research | Yes | Weighing of benefits and risks of genetic test results | Reference to the organization ‘Stiftung PATH – Patient’s Tumor Bank of Hope’ and their associated tumor bank as an offspring of mamazone e. V. |
| 8 | BRCA Netzwerk e. V.  https://www.brca-netzwerk.de/ | Breast Cancer | Not specified | Providing extensive information about diagnostic and operation possibilities, therapy and aftercare, promoting prevention, supporting affected persons | Yes | Weighing of benefits and risks of genetic testing.  References to counselling centers. | Organization publishes a regulary updated list of calls for participation on various research projects around breast cancer on the website |
| 9 | Stiftung PATH – Patient’s Tumor Bank of Hope  http://path-biobank.org/index.php/de/ | Breast Cancer | 10.628 donors (November 2017) | Increasing the chances of curing breast cancer | Yes | - | The organization has established a number of tumor banks located at various hospitals all over Germany for the collection of tumor tissue and blood samples of breast cancer patients. Tissue and blood samples are handed out to researchers on request after careful evaluation of research goals. |
| 10 | Netzwerk Männer mit Brustkrebs e. V.  https://www.brustkrebs-beim-mann.de/ | Breast Cancer in male individuals | Not specified | Supporting the interests of affected men in health policy | Yes | References to genetic aspects of the disease, references to counselling centers | Organization publishes a list of calls for participation on various research projects around breast cancer on the website |
| 11 | PKD Familiäre Zystennieren e. V.  http://www.pkdcure.de/ | Polycystic Kidney Disease | Not specified | Supporting research, public relations, and patient’s interests | Yes | Critical perspectives on genetic tests due to a lack of curative therapy options | Selected calls for participation in ongoing research projects are provided along with results of closed studies on the website |
| 12 | Deutsche Huntington-Hilfe e. V.  https://www.dhh-ev.de/ | Huntington’s Disease | 1644 | Self-help, sharing information about progresses in research, supporting regional self-help networks | Yes | Impact of genetic tests regarding possible psychosocial consequences are indicated, provision of a list of though-provoking impulses for persons interested in genetic testing, recommendation of counselling, references to various counselling centers | - |
| 13 | Niemann-Pick Selbsthilfegruppe Deutschland e. V.  https://www.niemann-pick.de/startseite.php | Niemann-Pick Disease | Not specified | Raising researchers awareness for the disease, improving collaborations, arranging genetic counseling for affected families | Yes | References to hereditary causes of the disease, access to genetic counselling for affected families as a goal of the organization | - |
| 14 | Tuberöse Sklerose Deutschland e. V.  https://www.tsdev.org/ | Tuberous Sclerosis | Not specified | Stimulating the exchange between patients, relatives, doctors, therapists, clinics and research institutes | Yes | Genetic causes of the disease are mentioned, though no direct references to genetic testing can be found | - |
| 15 | Cholesterin & Co. e. V.  https://cholco.org/ | Hypercholesterolemia and other hereditary disorders of the lipid metabolism | Not specified | Networking for the exchange of experiences, raising public awareness for the disease, stimulating research, stimulating international collaborations | Yes | Genetic causes of the disease are mentioned, recommendation of drawing a family tree in case that a hereditary passing on of the disease in the family is suspected | - |
| 16 | HOCM Deutschland e. V.  https://hocm.de/ | Hyperthrophic Obstructive Cardiomyopathy | 88 | Supporting research through participation, stimulating a dialogue among researchers | Yes | Information on hereditary aspects of disease is provided. | - |
| 17 | Mukoviszidose e. V.  https://www.muko.info/ | Cystic Fibrosis | Not specified | Increasing life expectancy and life quality of affected persons | Yes | References to genetic tests as a possibility to optimize therapy | Establishment of the German Cystic Fibrosis Registey |
| 18 | Kuratorium Deutsche Altershilfe  https://kda.de/ | Dementia | Not specified | Developing and extending counseling, accompaniment and help for elderly persons, realizing their self-determination | Yes | Publication of an article around the ethical aspects of dementia prediction in the organization’s magazine “ProAlter”, edition 4/18 | - |
| 19 | ALZheimer-ETHik e. V. http://www.alzheimer-ethik.de/ | Dementia | Not specified | Protection from exclusion, violence, medical malpractice | Yes | - | - |
| 20 | Aktion Demenz e. V.  https://www.aktion-demenz.de/ | Dementia | Not specified | Increasing public commitment for affected persons | Yes | - | - |
| 21 | Demenz-Support Stuttgart gGmbH  https://www.demenz-support.de/home | Dementia | Not specified | Preparing results of current research about dementia for different target groups, involvement in practical research, evaluation and process support | Yes | - | - |
| 22 | Deutsche DepressionsLiga e. V.  https://www.depressionsliga.de/ | Depression | 677 (Mai 2018) | Representation of affected people’s interest | Yes | Information about genetic factors in depression are provided on the website | Organization publishes a regulary updated list of calls for participation on various research projects around depression |
| 23 | Deutsche Hochdruckliga e. V.  https://www.hochdruckliga.de/ | Hypertension | 6000 | Raising public awareness for the disease | Yes | - | - |
| 24 | Schlaganfall-Ring Schleswig-Holstein e. V.  https://www.schlaganfall-ring.de/ | Stroke | Not specified | Desire to take responsibility for acute stroke care in the region | Yes | - | - |
| 25 | Stiftung Deutsche Schlaganfall Hilfe  https://www.schlaganfall-hilfe.de/de/start/ | Stroke | Not specified | Representation of affected people’s interests, establishing a dialogue between people and institutions in the health care sector | Yes | - | - |
| 26 | Hämochomatose-Vereinigung Deutschland e. V. (Regionale Kontaktstelle)  http://www.haemochromatose.org/ | Haemochroma-tosis | 440 | Discussing questions that are relevant for patients, collaboration with clinics, doctors, therapists, organizations, health insurances etc. | Yes | References to the genetic causes of the diseases are given within information texts on the website  References to human genetic counselling centers are provided on the website | - |
| 27 | Interessengemeinschaft Sichelzellkrankheit und Thalassämie e. V.  http://www.ist-ev.org/ | Sickle Cell Disease and Thalassemia | 950 | Raising public and medical awareness for the disease | No | References to the genetic causes of the diseases are given within information texts on the website | - |
| 28 | *Brustkrebs Deutschland e. V.  https://brustkrebsdeutschland.de/ | Breast Cancer | - | - | - | - | - |
| 29 | *Deutsche Gesellschaft für Muskelkranke e. V. (DGM)  https://www.dgm.org/ | Neuromuscular Diseases | - | - | - | - | - |
| 30 | * Deutsche Alzheimer Gesellschaft e. V.  https://www.deutsche-alzheimer.de/ | Dementia | More than 130 associa-ted orga-nizations | Supporting re-search on demen-tia and dementia care options, initiating initia-tives in health and social policy | Yes | Publication of an infor-mation sheet around the genetic aspects of Alz-heimer’s disease including genetic tests | - |

* Drop-out due to rejection of participation in the study

Annotation: Green highlighted organizations were selected based on findings from the NAKOS-database while grey highlighted organizations were selected through an additional web research focusing on a broader spectrum of organizations. White highlighted organizations rejected participation in the study.

1. Corresponding author: c.rauter@stud.uni-goettingen.de [↑](#footnote-ref-1)
2. [↑](#footnote-ref-2)
