# Supplementary material for: My Data, My Choice? – German Patient Organizations’ Attitudes towards Big Data-Driven Approaches in Personalized Medicine. An Empirical-Ethical Study
Source: J Med Syst. 2021 Feb 22;45(4):43. doi: 10.1007/s10916-020-01702-7 (PMC7900081; doi:10.1007/s10916-020-01702-7)

Suppl. 2: Interview guidelines

Title: My data, my choice? – German patient organizations’ attitudes towards Big Data-driven approaches in personalized medicine. An empirical-ethical study.

Journal: Journal of Medical Systems

Authors: Carolin Martina Rauter , Sabine Wöhlke, Silke Schicktanz

Affilation: Institute of Medical Ethics and History of Medicine, University Medical Center Göttingen, Humboldtallee 36, 37073 Göttingen


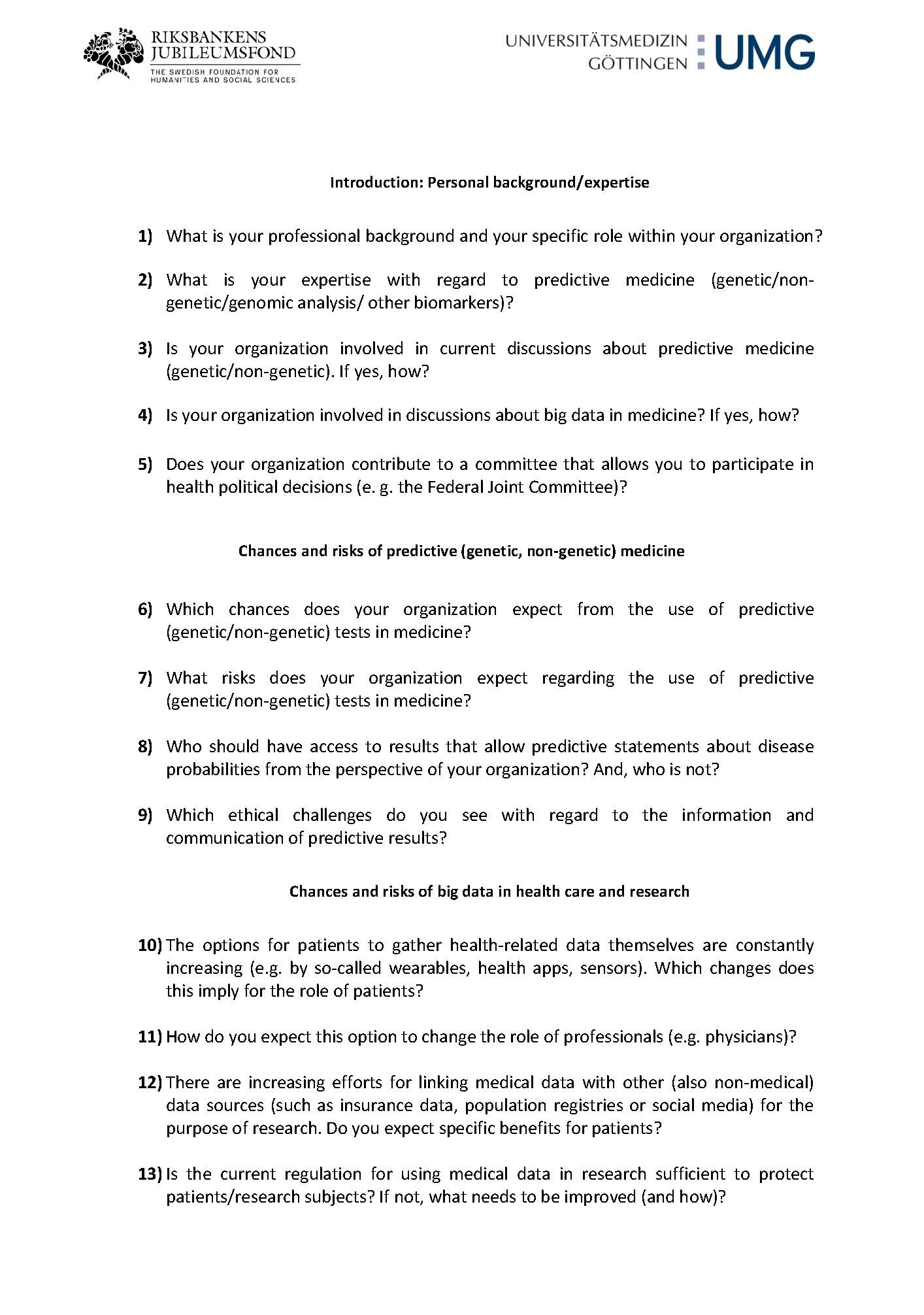


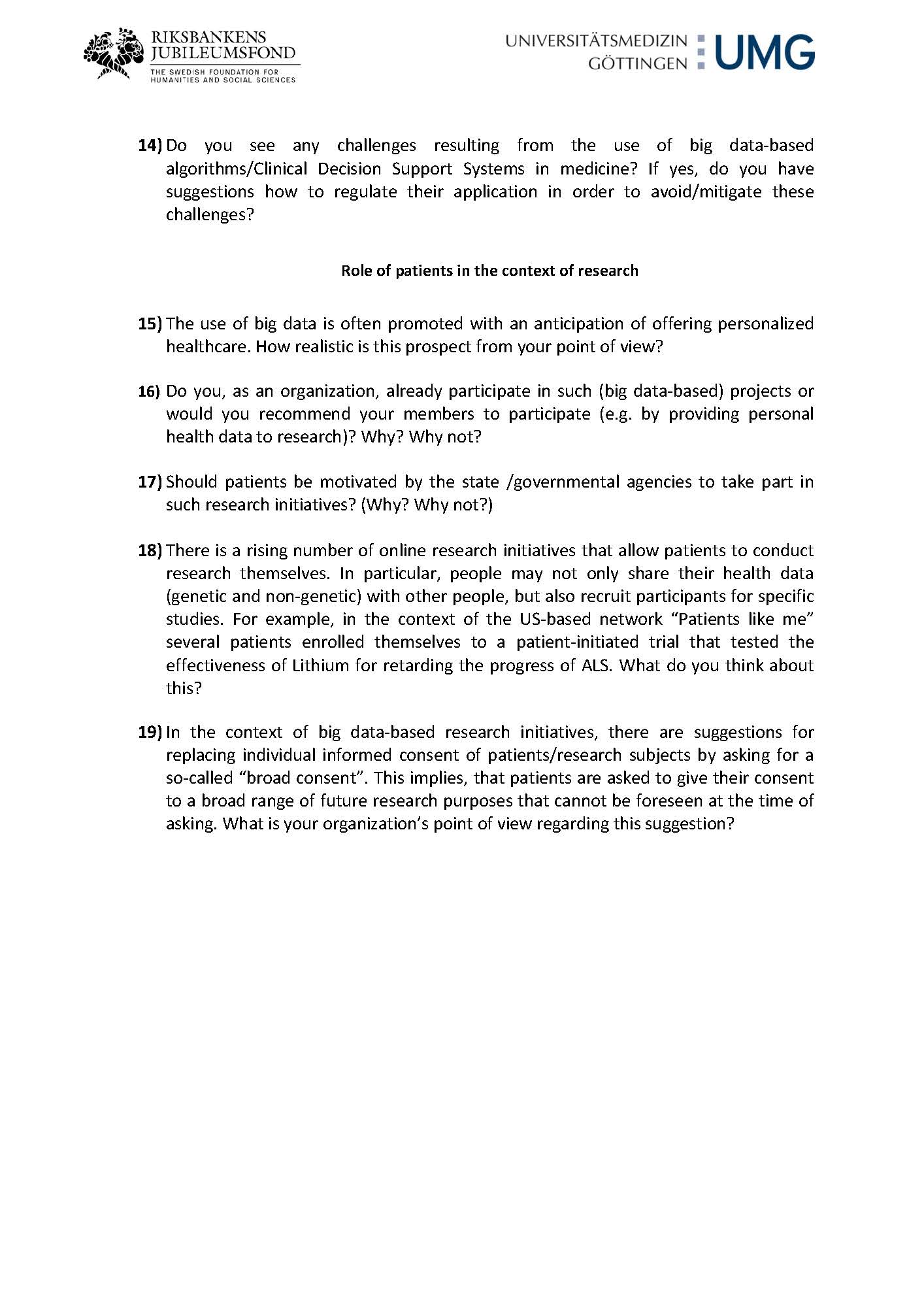

Supplement: Supplementary file 2 — (DOCX 491 kb) [file 10916_2020_1702_MOESM2_ESM.docx]
