# Supplementary material for: My Data, My Choice? – German Patient Organizations’ Attitudes towards Big Data-Driven Approaches in Personalized Medicine. An Empirical-Ethical Study
Source: J Med Syst. 2021 Feb 22;45(4):43. doi: 10.1007/s10916-020-01702-7 (PMC7900081; doi:10.1007/s10916-020-01702-7)
Supplement: Supplementary file 3 — (DOCX 56.8 kb) [file 10916_2020_1702_MOESM3_ESM.docx]

ESM Suppl. 3: Visualization of results from website analysis

Title: My data, my choice? – German patient organizations’ attitudes towards Big Data-driven approaches in personalized medicine. An empirical-ethical study.

Journal: Journal of Medical Systems

Authors: Carolin Martina Rauter^[[1]](#footnote-1)^, Sabine Wöhlke, Silke Schicktanz

Affilation: Institute of Medical Ethics and History of Medicine, University Medical Center Göttingen, Humboldtallee 36, 37073 Göttingen

Table 3: Examples of POs perspectives on benefits and risks of genetic testing and testing results – selected citations from 3 POs from website analysis:

| **Patient organization** | **Quote** | **Reference** | **Language** | **Accessed** |
| --- | --- | --- | --- | --- |
| mamazone e. V. – Forschung und Frauen gegen Brustkrebs | **Psychsocoial burden:** (…) „In case that multiple cases of breast and ovarian cancer have occurred in your family, it is recommended to consciously consider whether obtaining knowledge about having an inherited mutation is truly desired. Making a right decision in this case is not easy. Anyway, it remains a psychological burden to live with the knowledge of being confirmedly at risk.” (…)  **Participation in screenings:** (…) “Women at risk are given the opportunity to participate in more regular standardized check-ups than women at average risk in the general public.” (…)  **Opportunities for therapy optimization:** (…) Hopefully, targeted therapies will be developed and implemented in the future to increase the number of therapeutic options particulary for gene carriers.(…) | https://www.mamazone.de/brustkrebswissen/frueherkennung-diagnose/erblicher-brustkrebs/fragen-antworten/ | German  (own translation to English) | 11 Jan 2021 |
| Deutsche ILCO e. V. | **Options for prevention and therapy:** (…) “Roughly 3.500 persons annually are likely to suffer from hereditary forms of colon and colorectal cancer in Germany – a number far too high which leads to the conclusion that most people are not aware of their risk or refuse to apply their behavior to it. Also in these families benign precancerous stages (polyps) can almost always be identified during early detection and screening examinations. (…) | https://www.ilco.de/fileadmin/user_upload/Moeslein_IP_4_09.pdf | German (own translation to English) | 11 Jan 2021 |
| Verein VHL (von-Hippel-Lindau) betroffener Familien e. V. | **Accurate evaluation of test results required for sufficient validity:** (…) Subsequently to genetic counselling, a genetic test can be performed. In this context blood is screened for possible VHL-gene variants. It is very important to engage an experienced laboratory in this examination to receive a sufficiently interpreted and valid test result. (…)  **Decrease of psychosocial burden due to negative test results:** (…) If the test proves that no VHL disease runs in the family, no further examinations are required. Also for other family members, no further action is needed. | https://www.hippel-lindau.de/downloads/VHLflyer_Gendiagnostik.pdf | German  (own translation to English) | 11 Jan 2021 |

1. Corresponding author: c.rauter@stud.uni-goettingen.de [↑](#footnote-ref-1)
