# Supplementary material for: My Data, My Choice? – German Patient Organizations’ Attitudes towards Big Data-Driven Approaches in Personalized Medicine. An Empirical-Ethical Study
Source: J Med Syst. 2021 Feb 22;45(4):43. doi: 10.1007/s10916-020-01702-7 (PMC7900081; doi:10.1007/s10916-020-01702-7)
Supplement: Supplementary file 4 — (DOCX 57.6 kb) [file 10916_2020_1702_MOESM4_ESM.docx]

ESM Suppl. 4: Table 4: Quotations from semi-structured expert interviews with POs

Title: My data, my choice? – German patient organizations’ attitudes towards Big Data-driven approaches in personalized medicine. An empirical-ethical study.

Journal: Journal of Medical Systems

Authors: Carolin Martina Rauter, Sabine Wöhlke, Silke Schicktanz

Affilation: Institute of Medical Ethics and History of Medicine, University Medical Center Göttingen, Humboldtallee 36, 37073 Göttingen

|  | **Pseudonym** | **Quote (translated)** |
| --- | --- | --- |
| 1 | SE2WB | “I struggle with answering this question as I am not familiar with these predictive tests and, as I said, we have not been discussing them much. [M1: Mhm.] Well I.. uhh, well I.. It is hard for me to understand how they could be helpful for us regarding our argumentation. [M1: Yes.] I lack knowledge about that.” |
| 2 | SE2MB | „(…) We do tell our members that within the last 30 years, if you had asked experts, they would have said ‘In ten years, gene therapy will be available.’ If you ask them ten years later, they would again say ‘In ten years, gene therapy will be available.’ Eh, in the meantime it is said ‘Gene therapy will be available in four to five years.’ So, estimated time has shortened down, this is what we inform about, that all of this is about to be implemented.. Uhh, but without, as these things are not definite yet, saying ‘This is something.’ or providing any sort of counselling or something similar, as these things are just not definite yet.” |
| 3 | SE5MB | “(…) But I don’t think that, uhh, that if you’re diagnosed during an ultrasound examination ‘You have disease 14.’ then you’re right afterwards offered ‘We’ll take a look into the genes.’ No. Well, this is something I can’t.. relate to. No. Well, it is also not reported. (…) |
| 4 | SE2MB | “(…) Not with genetic tests. With gene therapy, uhh, surely. This will be established through virus vectors which, once implanted, depending on, whether they are young people…uuhm, these are new procedures. In medicine, there’s always the question: What is going to happen in the next thirteen, twenty, thirty, forty years, will this bear any consequences which can’t be foreseen at the moment? Which is why I believe that patients will approach this with a wait-and-see attitude.” |
| 5 | VK5MNB1 | (interrupts) “Well (unintelligible) and sympt-, eh, symptom 1 is a field in which many things are still at an early stage of development. So nothing can really be said about this. It was frequently reported that somewhat targets have been identified but symptom 1 is so complex that I assume that there is currently nothing really happening in the field of genetics. I am not entirely sure whether uhh, there has been this hype three years ago where it was said ‘Just send us a drop of your blood. It is sufficient when you drop it on a piece of paper and we, having a large data base, will provide a suitable statement on it.’ So, I think, regardless of the person promoting it in land 5, they must be dreamers or so. Thinking that there is already something going on is just a lie. (…) By looking closer, the topic of prediction is very thinned out.” |
| 6 | SE11WB | “And uhh, there are also many many considerations, especially overseas [M1: Mhm.], around gene therapy. But this is where one currently leans out of the window [M1: Yes.] because it is still, well, when talking about gene therapy now, it is still about experimental animals getting cancer and uhh, just to give an illustration for side-effects. [M1: Yes. Yes.] And uhh, this is…We currently talk about it, this is out of question [M1: Mhm.] but, in my point of view it is (sighs).. There has not been enough progress to fuel hopes. I am always a little down to earth and have to assess what is really there. [M1: Yes.] (…) But gene therapy still remains a difficult topic.” |
| 7 | SE10MB | “Eh, our disease is genetically determined. And uhh, there’s gene defect 5. It was discovered in 1993. [M1: Mhm.] And eh, shortly after that, there was the opportunity to have a genetic test. [M1: Mhm.] Uhh, we as an organization support this uhh.. when families, where disease 29 is suspected, want to get tested. This is due to.. uhh, early detection, so the annual screening for new tumors and the monitoring of tumor growth is the only sort of prevention we can do. [M1: Mhm.] And when you know that you’re affected by disease 29, you can undergo these clinical examinations, or at least it makes sense to do so. If you’re not affected, then children and young adults don’t have to undergo these procedures. [M1: Mhm.] So you’re only examining those in need of screening. [M1: Mhm.] And this is why we support genetic testing. And I believe that eh, 90 percent, maybe fiv-.. well, I would say definitely 90 percent, if not more, have themselves tested. (…)” |
| 8 | SE12MB | “Eh no, this is indeed of importance for us, for example, when you receive treatment and it’s about whether you should receive chemotherapy or not. This is a very important question. And it’s not only about that. At the moment, the situation is like eh, regarding prognosis, if your prognosis is bad, you receive chemotherapy. But regarding prediction, nothing is really clear. Eh, and everyone believes that this is the right treatment although you only know that your condition is a malign or a progressed disease 24. You don’t know how you’ll respond to treatment and this is the crucial part. And there are current aims to make improved statements about prognosis and therapy response based on gene analyses. And this is crucially important.” |
| 9 | SE7MB | (takes a deep breath) “Well, yes. This is again an ethical issue. Well, technically it is possible. There are two gene variants, gene defect 3 and gene defect 4. They can be identified perfectly. Then there are, how can I say, gene parts that can help to assess the speed of progression, so to say. [M1: Mhm.] From our view today, this does not or at least hardly make sense to us from an ethical point of view because it won’t lead to a change in therapy. Therapy would be identical anyway, whether I have been genetically tested or not. From our point of view, there are very few ethical reasons to have such a test and we have already stated them.” |
| 10 | SE9WB | “As I already said, it is a disease running in the family, a hereditary disease [M1: Mhm.]. And frequently, affected persons already have pictures in their heads. [M1: Mhm.] Pictures of more intensely affected people, either from their families or meeting other people. And uhh, also reflecting upon yourself by seeing more intensely affected people at hospitals, or centers specialized in disease 28. There are also videos on YouTube, terrible videos, illustrating the terminal stages of the disease. [M1: Yes.] These are no nice pictures.” |
| 11 | SE6WB | “During genetic testing? [M1: Mhm.] Uhh, well, another important aspect is that they’ll make you transparent, right? You’re a transparent patient then. Eh, personally, I don’t have a problem with this, it just comes to my mind, right? [M1: Mhm.] So there are people who might not want to know the diagnosis themselves. [M1: Mhm.] Right? Therefore in that sense, your ‘right-not-to-know’ about the disease is taken away from you in some way.(…) |
| 12 | VK1MNB8 | “If the validity of these tests could be ensured, they might bear opportunities. But I say this is not the case. So, if a likelihood or a risk could be assessed by a test, then it would bear the chance to take charge of yourself and plan ahead. (…)” |
| 13 | SE5MB | “Uhh, this won’t be stoppable. It will happen. Right? About this…I am one hundred percent sure. Also about these genetic tests. [M1: Yes.] Eh, even today you can already see, we’ve been talking about this before, if I take out a life insurance and there’s a question about previous genetic tests and if I give a false response and uhh, uhh…health insurance or the, the insurance company can refuse to pay for my eventual treatment. [M1: Mhm.] Uhh, for the patient, this is a conflict. On the one hand, I might have an interest in having the result but on the other hand I don’t want to have any disadvantages due to having a testing as well. [M1: Of course mhm.] Moreover it’s the same with health data gathered in this context. [M1: Mhm.] Right? It can always turn into disadvantages. And uhh, I am not sure whether all players involved have the same interests as the patient [M1: Mhm.].” |
| 14 | SE6WB | “(…) Then our child responded ‘Yes, there has been a genetic test.‘ and was asked about the current address of her doctor and they (annotation by the author: the employer) also requested the current medical reports. [M1: Mmh.] They were sent out to him and immediately afterwards, our daughter received a rejection. She was not even invited to the assessment center. Based on the diagnosis, she was rejected immediately. (…)” |
| 15 | VK3WB1 | “Me personally? [M1: Mhm.] Well, of course I know that knowledge regarding the insights of genetic mutations is only possible through good data sets. Therefore, it is important to read, collect and evaluate data in large amounts, so you can recognize deviations and their effects. [M1: Mhm.] So far, I know of course that the insights that help us to recognize and assess our risk rely on this. (…) And uhh (unintelligible sentence) all of these drugs that are developed now. This is only possible through many data sets that indicate ‘Well, with this or that feature this and that works, this or that does not work.’ [M1: Mhm.] For this, probably even larger data sets are needed to be processed. (…)” |
| 16 | SE9WB | “Uhh.. no. Well, I see it rather as an opportunity. [M1: Mhm.] What I am thinking of is an observation study that is currently running around disease 28 where information is accumulated over many years and evaluated subsequently. [M1: Mhm.] Uhh and which leads to connections that couldn’t be made without the large amounts of data included. [M1: Mhm.] For example, people suffering from disease 28 smoke a lot. And if you can arrive at conclusions about other characteristics that affected people have in common [M1: Mhm.], for example a gene that has not yet been discovered. [M1: Mhm.] And we say ‘Okay, the heavy smokers have, I don’t know, gene x and y. Then it is said, ‘Okay, we should investigate on this.’ [M1: Mhm.] (…) So for me, this is, on the contrary, a great opportunity.” |
| 17 | SE1MB | “Yes, I do believe that. Uhh, we already see that in some fields around disease 1. For example, treatment of seizures will be different in the future uhh, as based on data analysis and analyses of the patient uhh, drugs can be dosed differently which would lead to a reduction of overdoses and along with that also a possible decrease in side effects. I can approach this issue more on an individual level and the production of drugs produced for individuals will surely be a result of that. We have a quite positive attitude on this.” |
| 18 | SE8MNB | (sighs) (5 seconds break) “Well, I think that... there are more and more drugs, also for disease 27, that are, so to speak, designed specifically for these patients. [M1: Mhm.] And this could probably not happen without the available data. [M1: Mhm.] Because, you can get insights through these amounts of data that you probably wouldn’t be able to get by referring only either to individual cases or just singular countries. [M1: Mhm.] And therefore, I believe that it is useful.” |
| 19 | VK3WB1: | “Well, I do believe that this can also lead to certain benefits. Maybe it will improve the identification of risk factors. Uhh, all of this I can imagine.. To approach the issue more specifically. (…)” |
| 20 | SE7MB | “(...) Well, in our field, disease 26 [M1: Mhm.], the case is unfortunately.. Or, let’s take up another example, say dialysis patients. Five or six years ago, there has been a dialysis register. It provided exact details of how many dialysis patients there are uhh, how old they are, how good or bad their treatment were [M1: Mhm.]. And today, it’s a blind flight.. There is no information available on how many dialysis patients there are. You have to assess that with an enormous effort through the health insurance funds now. [M1: Mmh.] Therefore, I see huge disadvantages if registers are not available. Good medicine requires that registers are available, that statistical relevant patient data is available for evaluation.” |
| 21 | SE2WB | “Well, I would say that those who use it really appreciate it. Well I.. They use it because it facilitates the process of documentation tremendously. In the beginning, it was.. The systems have surely developed [M1: Mhm.]. Uhh, meanwhile it became available for smartphones, through a smartphone-app. And, uhh, for example the batch number is saved. Only small things but.. In the beginning, you had to add it each time and in the meantime it is saved. And this way it’s all faster. (…) Uhh, and this makes the whole handling easier, although some people still do paper documentation as well.” |
| 22 | SE11WB | “Well, I think that now, if for example..uhh,, well, if I imagine now that I add my data to this and then the responsible doctor can have a look at it [M1: Mhm.] Like this, right? Do we have to imagine it like that? Yes. We can do this, for example. Uhh, then it’s probably a huge facilitation to have uhh, like a pool, where his or her patients so to say.. Or if I think about having five additional specialized doctors and if they have to get updated about everything. If there was such a pool, where he or she could have filed everything, then I think the doctor would only have to look at it for two minutes and then the patient record was online, for example. [M1: Mhm.] And I think this is a tremendous facilitation for doctors.” |
| 23 | SE2MB | “No (snorts assessing). That’s possible too, but this is less.. Well... Let’s say in the history of disease 3, those who did better on their record during the scandal around infection 1 had better chances to receive compensations as they were more capable of proving which charges they had received. Then, it was more likely to find out whether the received charge was infected. And by this way, they were able to prove that they got the infection this way. [M1: Mhm.] Right? And those, who did worse on their record had more trouble.” |
| 24 | SE4WB | “Well, I go to the gym on a regular basis and uhh.. I see that when I am running on the treadmill or riding the bike, like my pulse and uhh, such things and uhh, this important for me.” |
| 25 | SE4WB | “Mhm, yes, and also preventative, so I am aware that none of my bodily functions worses but that everything is alright.” |
| 26 | SE12MB | “Yes, yes, of course. [M1: Mhm.] I can comprehend at any given time, or.. I can comprehend many things regarding my own health. It starts with the heart rate and surely continues with the blood pressure and such things. And uhh, of course it is beneficial for me, right, if I learn more about my own health. [M1: Mhm.] And if you could extend that to, for example, I mean I do not suffer from disease 2 but I can imagine that for people suffering from disease 2, it would be beneficial for their health to see what their medical parameter 10 is by using an app. But as I said, then the danger of your data getting harvested is something that you have to live with.” |
| 27 | SE10MB | “I haven’t made up my mind about this yet. It is, well, it is not specific for our disease that we.. track something like that. Uhh, if someone is doing this individually for him- or herself, yes, then the person should do it. Uhh, however, he or she has to be conscious about that uhh, those who might be receiving this data, right? [M1: Mhm.] But uhh, I haven’t uhh.. I don’t own something like that. I have uhh, I have a step counter on my smartphone, an app (laughs) eh, with a step counter but nothing more. [M1: Mhm.] And uhh, I haven’t made up my mind about it yet. Especially not with reference to the issues of self-help.” |
| 28 | SE2WB | “(...) And I would say that our patients are rather open to this, uhh because, I’d say that if it leads to any benefits for them at the end of the day, they’ll work with it [M1: Mhm.]. Now I have to think. I think there is uhh, a project of the university in city 6 [M1: Mhm.], project 1.[M1: Mhm.] And they offer a so called project 1-camp two times a year. I believe that during this camp, the patients wear fitness trackers. [M1: Mhm.] I am quite sure about it. So those who are participating in this are absolutely willing to..Well, let’s say, I believe these are parts of multiple studies that are running as part of this project. [M1: Mhm.] Uhh, this has been running for a few years now. Uhh, they naturally collect many data to generate study results. [M1: Yes.] Uhh, and as I said, the participants, they are all willing to be walking around with these fitness trackers.” |
| 29 | VK4WB1 | “Uhh, I see uhh, an extremely huge potential for Big Data in health care research [M1: Mhm.] As long as it is based on the patient’s informed consent, speaking of data donation.” |
| 30 | SE10MB | “Well, I do not tend to see any use in this. I rather see the danger in it, to be honest. Because, if I look around who is posting what on Facebook which is somehow linked to the disease and.. I even think that this happens. I see this rather uhh…critical. More critical than useful, right? So if Facebook or Google know that you are suffering from disease x or y because you access an associated website frequently, you’re posting things that you’re in a hospital or that you’re undergoing surgery or whatever. Uhh, I have a critical attitude about this [M1: Mhm.]. “ |
| 31 | SE9WB | “I see the danger of misuse or that the patient is not well informed or that he/she does not know what the data will be used for. [M1: Mhm.] This is, for me, the crucial point, uhh, knowing what is measured and what is not, what is happening with my data, how it is saved, with my name, my address, uhh, or if it is saved under a pseudonym, a number or something like that and who has which kind of access to it later on. [M1: Mhm.] Eh, if I save everything, including geographical data, then conclusions can be made that are not useful.” |
| 32 | SE10MB | “(...) And uhh, this is very speculative but uhh, we have already experienced euthanasia of persons whose lives were considered to be unworthy under the Nazi regime. Now, think about a totalitarian state desiring that all people affected by hereditary cancer or another disease shall not have children anymore. [M1: Mhm.] This is uhh, very speculative. But uhh, of course, this idea is of a totally different quality, right?” |
| 33 | SE12MB | “Yes, workload can be reduced, but as I said.. it may lead to the treatment of diseases rather than human beings, meaning that every treatment includes possible side effects. And uhh…the success of a treatment has to be balanced against these side effects. [M1: Mhm.] Uhh, and this process of evaluation can only be made face to face in a conversation with the patient, not with algorithms or things like that. [M1: Mhm.] And there’s always the danger of treating the disease rather than the individual.“ |
| 34 | VK2MNB1 | “It (annotation: the data) has to be the patients‘ property. Any other definition is doomed to failure. [M3: Well, alright but..] At the very moment, at which a patient does not own his or her data and cannot create an authorization concept, which he or she can of course adjust at any time, this soon may get to a critical point, right? [M3: Mhm.] And uhh, we don’t have to look at country 5, to Google and Facebook and so on because there are already some efforts in Germany where those who aim to take care of patient records, uhh, where you get the impression that it is all about supply space.“ |
| 35 | SE12MB | “I see a problem when patients have become sick a short time ago [M1: Mhm.] that they may uhh.. be in a in a special emotional state and may consent to something they might have negotiated at a later point in time. [M1: Mhm.] And uhh, this might become a huge problem because it takes effort to collect data. Blood tests or other tests might be needed. [M1: Mhm.] Uhh, and if an objection is raised and you can throw everything away afterwards, then all the effort was nothing good for, right?“ |
| 36 | SE1MB | “I imagine that much data will be collected in the future and uhh.. in this case, rules for the anonymization and uhh.. collection of untraceable data have to be figured out.“ |
| 37 | SE1MB | “Yes, in the public discussion around data protection and Big Data, uhh, although it is not named this way, there is an overall scpeticism in the general public regarding the confidentiality of medical data. We try to counteract against this because naturally we have an interest in patients participating in trials and associated surveys. Uhh, if data is anonymized and pseudonymized, then hurdles are not so high and the rate of patients participating is quite good.“ |
| 38 | SE12MB | “Uhh, you can do all kinds of possible things with this data. Particularly in the case of rare diseases, if you have someone who is affected by disease 35 and he or she has another uhh.. characteristic, then of course you can backtrack to the individual person, right? Let’s say, I am an air traffic controller. [M1: Mhm.] And then you go out and say ‚There has been an air traffic controller.‘ because you may include factors such as professional backgrounds, uhh, exposition to poisonous substances or radiation or whatever is important in your area of research, then all over Germany, there would probably be no other person except me who has these two characteristics. I don’t know, maybe there’s a second or a third person, right? [M1: Mhm.] But as it is a rare disease and also air traffic controller is rather a rare profession, uhh.. this is something where you can anonymize as much information as you want to, if you have these two or three characteristics, then it may be possible to backtrack to an individual person very quickly. This is something used in dragnet investigations as well. [M1: Mhm.] And this is something where I see a problem, especially if I have a look at all those countries in which democracy is impeded. (…)“ |
| 39 | SE2MB | “This was something we have been fighting for by saying ‚If this will be implemented, it has to be worked out according to data protection regulations.’ We have always buckled down to this to make sure it is approved by the data protection officers.“ |
| 40 | SE12MB | “Well, I am aware of the fact that every data base can be hacked, right? [M1: Mhm.] That’s why.. I know for example that clinic 1 has designed an isolated computer system for disease 3 that is not connected to the internet. [M1: Mhm.] To protect that. Uhh..you have to protect such things in a very good way. Aside of that I have no concerns.“ |
| 41 | SE7MB | “I think the interlinkage between registries of universities is bad, uhh.. the interlinkage between universities is often not implemented. There are often isolated solutions for certain things and if you have such a registry, normally everyone owing such a data source should be able to integrate the data for the benefit of everyone. But I think this is a problem in Germany. In other countries, I think researchers are connected in better ways.“ |
| 42 | SE1MB | “Well, of course we participate in all kinds of healing attempts, we support the recruitment of participants for trials covering a broad spectrum also aside of genetic testing. These aim for example to investigate in drug compatibility, side effects and also efficacy of treatment.“ |
| 43 | SE1MB | “Well, on the one hand, patients are also sceptical when data is collected by us so we also have to state sufficient reasons when we do something like that. [M1: Mmh.] And eh, on the other hand we also have other research projects that we support, which means that we have to make use of funds in a conscoius way. At the moment, I assume that roughly a third of the funding that we raise through our foundation is used for data collection etc.“ |
| 44 | SE2WB | “(…) At the moment, we are sponsored very well. But, of course you have to admit that we benefit from the good financial situation of the industry. If this would change significantly, then it would be far more difficult to finance the work of our association. (…) And we receive.. Of course we receive donations by.. Well, hardly of anyone else who is not.. industrially linked to us and has interests. [M1: Mhm. Thank you.] So the financial equipment is naturally a crucial factor for making our work possible.“ |
| 45 | SE5MB | “Uhh.. once the question about automatic debit was raised and if it makes sense for us, fiscal aspects like.. Which software is available on the market that you can use as an association? [M1: Mhm.] Right? And of course this is a question of money, Which investments do I have to make as an association [M1: Yes.] with no great financial resources at our disposal.“ |
| 46 | SE8MNB | “(…) It is about certifying, about developing medical guide lines, we operate our own registry [M1: Mhm.], uhh, we coordinate a trial network. (…)“ |
| 47 | SE10MB | “(…) This means uhh.. We own a biobank in which we store tumor tissue, We have our own registry in which affected people, I mean no doctor but the patients themselves, can enter their medical history on their own. (…)“ |
| 48 | SE8MNB | “Uhh, well, we have a registry and we receive a lot of requests in this context. Everyone wants to know something. Well, you can’t just call us and say ‚I want to know x and y.‘, there are application procedures and this also has to be paid partly. (…) |
| 49 | SE2WB | “(…) We ourselves as a patient association, eh, we do not collect any data. Well, we conduct surveys from time to time, but nevertheless, this is not very close to Big Data. (…)“ |
| 50 | SE2WB | “(…) But these are, let’s say, results of data that are also very important and interesting for us, Because this way, we can argue towards political representatives in a different way regarding treatment, treatment situations, compensations et cetera, so basically the whole politicial lobbying that we have to do to ensure therapy quality.“ |
| 51 | VK1WB5 | “So the professional background is that I became aquainted with the topic when I was affected myself. There has been a diagnosis, mid-stage disease 42. I had fantastic doctors (…) who took a different path with me (…). [M3: Mmh.] Uhh, and this, this is where it all started and eventually a self help group developed out of it“ |
| 52 | SE1MB | “It is very intense, also workwise, for colleagues from our executive board who take charge of this as well as doctors. It is a contact on eye level. This is not a matter of course in the self help environment. It is a very grown community where you get along well with eachother also on a personal level which is enormously helpful for such projects.“ |
| 53 | SE12MB | “Of course I wish for a better cooperation with the professional side, right? [M1: Yes. Mhm.] For people who are interested in this topic and you often notice that there are people being punctually interested. However, they are so involved in their medical daily routines that their commitment usually remains very punctual and short term and nobody really sticks to the topic for a longer period. Yes.“ |
